# Crystal structure of 4,8-di-*tert*-butyl-6,6-di­chloro-13-ethyl-2,10-dimethyl-13,14-di­hydro-12*H*-dibenzo[*d*,*i*][1,3,7,2]dioxaza­silecine toluene 0.25-solvate

**DOI:** 10.1107/S2056989015023889

**Published:** 2015-12-16

**Authors:** Ekaterina A. Kuchuk, Kirill V. Zaitsev, Sergey S. Karlov, Mikhail P. Egorov, Andrei V. Churakov

**Affiliations:** a N.D. Zelinsky Institute of Organic Chemistry, Leninsky prospekt 47, Moscow 119991, Russian Federation; b Department of Chemistry, M.V. Lomonosov Moscow State, University, Moscow, Russian Federation; c Institute of General and Inorganic Chemistry, Russian Academy of Sciences, Moscow, Russian Federation

**Keywords:** heavy carbenes, penta­coordinated silicon, crystal structure

## Abstract

The coordination polyhedron at the silicon atom in the title compound, C_26_H_37_Cl_2_NO_2_Si·0.25C_7_H_8_, is typical for penta­coordinated silicon derivatives and represents a slightly distorted trigonal bipyramid with an N atom and a Cl atom in the apical positions and the two O atoms and the other Cl atom occupying the equatorial sites. There are two independent mol­ecules in the asymmetric unit. The N–Si–Cl fragment in each is close to linear [178.24 (5) and 178.71 (5)°], in good agreement with 4*e*–3*c* theory, as is the elongation of the apical bond lengths [Si—Cl = 2.1663 (7) and 2.1797 (7) Å] in comparison with the equatorial bonds [Si—Cl = 2.0784 (7) and 2.0748 (7) Å]. Orthogonal least-squares fitting of the two independent mol­ecules resulted in r.m.s. deviation of 0.017 Å. The conformations of the two mol­ecules are almost the same, with corresponding torsion angles differing by less than 5.5°. The toluene solvent mol­ecule is disordered about an inversion centre.

## Related literature   

For general background to the chemistry affording the 2,2-[(alkyl­imino)­dimethanedi­yl]diphenols as ligands, see: Wichmann *et al.* (2012 ▸). For hypervalent silicon compounds, see: Holmes (1996 ▸); Rendler & Oestreich (2005 ▸); Selina *et al.* (2006 ▸). The title compound was obtained as part of our study of the ability of different types of tridentate ligands to stabilize ‘heavier carbenes’, see: Huang *et al.* (2012 ▸, 2013 ▸); Kireenko *et al.* (2013 ▸).
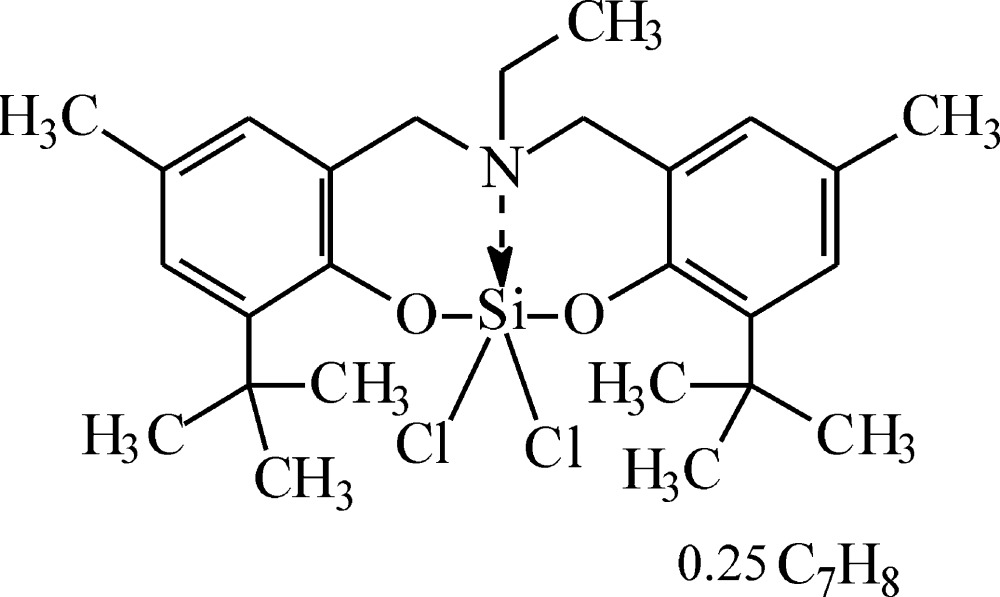


## Experimental   

### Crystal data  

2C_26_H_37_Cl_2_NO_2_Si·0.5C_7_H_8_
*M* *_r_* = 1035.18
Triclinic, 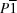

*a* = 13.9017 (9) Å
*b* = 13.9542 (9) Å
*c* = 16.9827 (11) Å
α = 69.649 (1)°
β = 65.978 (1)°
γ = 83.585 (1)°
*V* = 2819.6 (3) Å^3^
*Z* = 2
Mo *K*α radiation
μ = 0.30 mm^−1^
*T* = 150 K
0.35 × 0.35 × 0.20 mm

### Data collection  

Bruker SMART APEXII diffractometer
Absorption correction: multi-scan (*SADABS*; Bruker, 2008 ▸) *T*_min_ = 0.903, *T*_max_ = 0.943
27366 measured reflections
12314 independent reflections
9442 reflections with *I* > 2σ(*I*)
*R*_int_ = 0.034

### Refinement  

*R*[*F* ^2^ > 2σ(*F* ^2^)] = 0.043
*wR*(*F* ^2^) = 0.109
*S* = 1.04
12314 reflections
659 parameters
15 restraints
H-atom parameters constrained
Δρ_max_ = 0.33 e Å^−3^
Δρ_min_ = −0.27 e Å^−3^

### 

Data collection: *APEX2* (Bruker, 2008 ▸); cell refinement: *SAINT* (Bruker, 2008 ▸); data reduction: *SAINT*; program(s) used to solve structure: *SHELXTL* (Sheldrick, 2008 ▸); program(s) used to refine structure: *SHELXTL*; molecular graphics: *SHELXTL*; software used to prepare material for publication: *SHELXTL*.

## Supplementary Material

Crystal structure: contains datablock(s) I. DOI: 10.1107/S2056989015023889/zp2021sup1.cif

Structure factors: contains datablock(s) I. DOI: 10.1107/S2056989015023889/zp2021Isup2.hkl

Supporting information file. DOI: 10.1107/S2056989015023889/zp2021Isup3.mol

Supporting information file. DOI: 10.1107/S2056989015023889/zp2021Isup4.cml

**Fig. 1.**
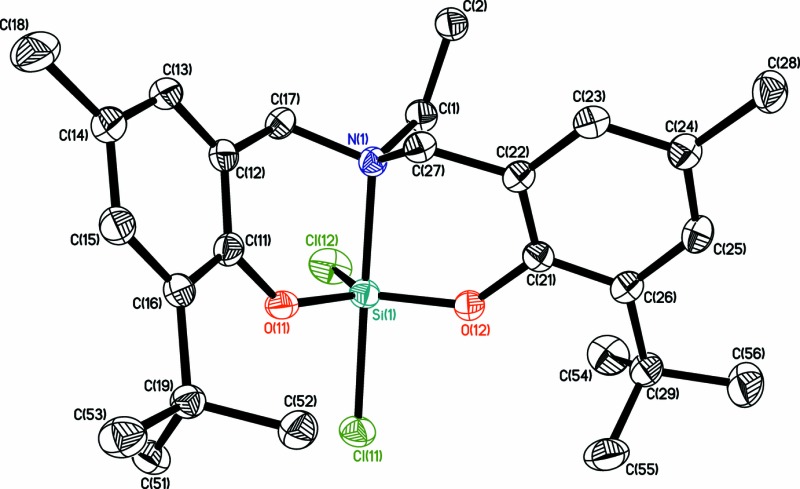
The molecular structure of one of the independent molecules of the title compound, with displacement ellipsoids shown at the 50% probability level. The toluene solvent molecule and hydrogen atoms are omitted for clarity.

. DOI: 10.1107/S2056989015023889/zp2021fig1.tif
The mol­ecular structure of one of the independent mol­ecules of the title compound, with displacement ellipsoids shown at the 50% probability level. The toluene solvent mol­ecule and hydrogen atoms are omitted for clarity.

CCDC reference: 1442032

Additional supporting information:  crystallographic information; 3D view; checkCIF report

## supplementary crystallographic information

### S1. Comment

The low valent derivatives of group 14 elements (Si, Ge, Sn) attract much attention because of interest in "heavier" carbon analogs. In general, silicon derivatives are highly reactive species, while germanium and tin analogs are more stable due to the known "inert pair" effect, but still demand for the additional stabilization. The stabilization of highly reactive "heavy carbene" centers may be accomplished using two approaches. The kinetic stabilization may be caused by the introduction of voluminous groups to the central atom; the thermodynamic stabilization may be achieved by donation of electron density from substituents to a vacant orbital of the central atom. As a part of our program to study the ability of the different types of tridentate ligands for stabilization of "heavier carbenes" (Kireenko *et al.*, 2013, Huang *et al.*, 2013, Huang *et al.*, 2012) we obtained and studied the structure of title compound, EtN{CH_2_[(5-Me)(3-^*t*^Bu)C_6_H_2_(−2-O)-}_2_SiCl_2_·0.25C_7_H_8_, which may be regarded as a promising compound for further reduction to prepare a silylene.

The structure of the title compound is shown on Fig. 1. Asymmetric unit contains two independent molecules with very close geometrical parameters. The orthogonal least-squares fitting of the two independent molecules resulted in root-mean-square deviation 0.017 Å. The conformations of these two molecules are almost the same since the corresponding torsion angles differ by less than 5.5 °. The coordination polyhedron at the silicon atom is typical for pentacoordinated silicon derivatives and represents a slightly distorted trigonal bipyramide with N(1) and Cl(11) atoms in apical positions and oxygen atoms O(11), O(12) and chlorine Cl(12) occupying equatorial sites. The N(1)—Si(1)—Cl(11) fragment is close to linearity (178.24 (5)°) that is in good agreement with 4 e-3c theory as well as the elongation of apical bond length Si(1)—Cl(11) 2.1663 (7) Å in comparison with that for equatorial bond (Si(1)—Cl(12) 2.0784 (7) Å). The N(1)—Si(1) distance (2.0452 (15) Å) lies within the standard range for related silicon species with electronegative substituents attached to the silicon atom (Selina *et al.*, 2006). The nitrogen atom has an approximately tetrahedral environment with bond angles ranging from 107.07 (10)–113.53 (11)° and is shifted towards the Si atom. In crystal, solvate toluene molecule lies on inversion centre. No classical hydrogen bonds are present between the host molecules or between host and guest molecules, while only weak intermolecular van der Waals interactions contribute to the stability of the crystal.

### S2. Experimental

The title compound was prepared with high yield from reaction of corresponding free ligand with tetrachlorosilane in presence of triethylamine as a base (two equivalents) in toluene solution at −20° C.

NMR spectra of title compound: ^1^H NMR (400 MHz, CDCl_3_, p.p.m.): δ = 0.98 (t, J=7.1 Hz, 3H, CH_2_C*H*_3_), 1.44 (s, 18H, C(C*H*_3_)_3_), 2.26 (s, 6H, C*H*_3_-Ar), 2.97 (br s, 2H, C*H*_2_ in Et), 3.98 (br s, 4H, NC*H*_2_Ar), 6.67 (br s, 2H, Ar), 7.10 (d, J=1.8 Hz, 2H, Ar).

^13^C NMR (100 MHz, CDCl_3_, p.p.m.): δ = 6.02 (CH_2_*C*H_3_), 20.84 (*C*H_3_—Ar), 29.36(C(*C*H_3_)_3_), 34.55(*C*(CH_3_)_3_), 47.99, 53.97 (*C*H_2_CH_3_ and N*C*H_2_Ar), 119.38, 126.24, 128.06, 131.11, 139.82, 149.12 (Ar).

^29^Si NMR (80 MHz, CDCl_3_, p.p.m.): δ = −123.94 (*s*).

Anal·Calc. for C_26_H_37_Cl_2_NO_2_Si (494.5690): C, 63.14; H, 7.54; N, 2.83. Found: C, 63.47; H, 7.86; N, 2.64%.

The crystals suitable for X-Ray analysis were grown from toluene/hexane solution.

### S3. Refinement

All non-hydrogen atoms were refined with anisotropic thermal parameters. Aromatic carbon atoms of solvent toluene molecule were refined with slightly restrained C—C distances (SADI). All hydrogen atoms were placed in calculated positions and refined using a riding model, with C—H = 0.95–0.99 Å, and with *U*_iso_(H) = 1.2 *U*_eq_(C) or 1.5 *U*_eq_(C) for methyl H atoms. A rotating model was applied to the methyl groups. Six outliers were omitted in the last cycles of refinement.

### Crystal data

**Table:**

2C_26_H_37_Cl_2_NO_2_Si·0.5C_7_H_8_	*Z* = 2
*M**_r_* = 1035.18	*F*(000) = 1106
Triclinic, *P*1	*D*_x_ = 1.219 Mg m^−^^3^
Hall symbol: -P 1	Mo *K*α radiation, λ = 0.71073 Å
*a* = 13.9017 (9) Å	Cell parameters from 5818 reflections
*b* = 13.9542 (9) Å	θ = 2.2–27.9°
*c* = 16.9827 (11) Å	µ = 0.30 mm^−^^1^
α = 69.649 (1)°	*T* = 150 K
β = 65.978 (1)°	Block, colourless
γ = 83.585 (1)°	0.35 × 0.35 × 0.20 mm
*V* = 2819.6 (3) Å^3^	

### Data collection

**Table:**

Bruker SMART APEXII diffractometer	12314 independent reflections
Radiation source: fine-focus sealed tube	9442 reflections with *I* > 2σ(*I*)
Graphite monochromator	*R*_int_ = 0.034
ω scans	θ_max_ = 27.0°, θ_min_ = 1.7°
Absorption correction: multi-scan (*SADABS*; Bruker, 2008)	*h* = −17→17
*T*_min_ = 0.903, *T*_max_ = 0.943	*k* = −17→17
27366 measured reflections	*l* = −21→21

### Refinement

**Table:**

Refinement on *F*^2^	Primary atom site location: structure-invariant direct methods
Least-squares matrix: full	Secondary atom site location: difference Fourier map
*R*[*F*^2^ > 2σ(*F*^2^)] = 0.043	Hydrogen site location: inferred from neighbouring sites
*wR*(*F*^2^) = 0.109	H-atom parameters constrained
*S* = 1.04	*w* = 1/[σ^2^(*F*_o_^2^) + (0.049*P*)^2^ + 0.4643*P*] where *P* = (*F*_o_^2^ + 2*F*_c_^2^)/3
12314 reflections	(Δ/σ)_max_ = 0.001
659 parameters	Δρ_max_ = 0.33 e Å^−^^3^
15 restraints	Δρ_min_ = −0.27 e Å^−^^3^

### Special details

**Table:**

Geometry. All e.s.d.'s (except the e.s.d. in the dihedral angle between two l.s. planes) are estimated using the full covariance matrix. The cell e.s.d.'s are taken into account individually in the estimation of e.s.d.'s in distances, angles and torsion angles; correlations between e.s.d.'s in cell parameters are only used when they are defined by crystal symmetry. An approximate (isotropic) treatment of cell e.s.d.'s is used for estimating e.s.d.'s involving l.s. planes.
Refinement. Refinement of *F*^2^ against ALL reflections. The weighted *R*-factor *wR* and goodness of fit *S* are based on *F*^2^, conventional *R*-factors *R* are based on *F*, with *F* set to zero for negative *F*^2^. The threshold expression of *F*^2^ > σ(*F*^2^) is used only for calculating *R*-factors(gt) *etc*. and is not relevant to the choice of reflections for refinement. *R*-factors based on *F*^2^ are statistically about twice as large as those based on *F*, and *R*- factors based on ALL data will be even larger.

### Fractional atomic coordinates and isotropic or equivalent isotropic displacement parameters (Å^2^)

**Table:**

	*x*	*y*	*z*	*U*_iso_*/*U*_eq_	Occ. (<1)
Si1	0.19585 (4)	0.68718 (4)	0.22126 (3)	0.02161 (12)	
Cl11	0.05925 (4)	0.58326 (4)	0.29544 (3)	0.02928 (12)	
Cl12	0.13951 (4)	0.76756 (4)	0.12122 (4)	0.03714 (13)	
O11	0.16095 (10)	0.72681 (9)	0.30901 (8)	0.0253 (3)	
O12	0.27134 (9)	0.58945 (9)	0.21036 (8)	0.0242 (3)	
N1	0.32674 (11)	0.78269 (11)	0.15403 (10)	0.0206 (3)	
C1	0.37796 (14)	0.79998 (14)	0.05230 (12)	0.0246 (4)	
H1A	0.3339	0.8464	0.0229	0.029*	
H1B	0.3783	0.7337	0.0430	0.029*	
C2	0.49005 (15)	0.84486 (15)	0.00403 (13)	0.0279 (4)	
H2A	0.5122	0.8637	−0.0622	0.042*	
H2B	0.5374	0.7940	0.0239	0.042*	
H2C	0.4928	0.9058	0.0193	0.042*	
C11	0.19693 (14)	0.79602 (14)	0.33347 (13)	0.0231 (4)	
C12	0.26165 (14)	0.87820 (13)	0.26483 (12)	0.0233 (4)	
C13	0.29633 (14)	0.94926 (14)	0.28856 (13)	0.0274 (4)	
H13	0.3407	1.0055	0.2419	0.033*	
C14	0.26703 (15)	0.93927 (15)	0.37945 (14)	0.0293 (4)	
C15	0.20453 (14)	0.85404 (14)	0.44615 (13)	0.0265 (4)	
H15	0.1860	0.8460	0.5085	0.032*	
C16	0.16763 (14)	0.77981 (14)	0.42635 (12)	0.0227 (4)	
C17	0.29669 (15)	0.88514 (13)	0.16676 (13)	0.0260 (4)	
H17A	0.3579	0.9338	0.1284	0.031*	
H17B	0.2390	0.9120	0.1460	0.031*	
C18	0.30313 (19)	1.01687 (18)	0.40609 (16)	0.0451 (6)	
H18A	0.3061	1.0852	0.3618	0.068*	
H18B	0.3733	1.0001	0.4066	0.068*	
H18C	0.2534	1.0157	0.4672	0.068*	
C19	0.09926 (14)	0.68634 (14)	0.50205 (12)	0.0232 (4)	
C21	0.37690 (14)	0.56835 (13)	0.18610 (12)	0.0214 (4)	
C22	0.44557 (14)	0.63868 (13)	0.17896 (11)	0.0201 (4)	
C23	0.55179 (14)	0.61776 (14)	0.15658 (12)	0.0227 (4)	
H23	0.5988	0.6662	0.1515	0.027*	
C24	0.58972 (14)	0.52621 (14)	0.14157 (12)	0.0239 (4)	
C25	0.51845 (15)	0.45813 (14)	0.14912 (12)	0.0255 (4)	
H25	0.5442	0.3957	0.1388	0.031*	
C26	0.41128 (14)	0.47537 (13)	0.17086 (12)	0.0224 (4)	
C27	0.40367 (14)	0.73555 (13)	0.19768 (12)	0.0211 (4)	
H27A	0.4630	0.7849	0.1738	0.025*	
H27B	0.3682	0.7209	0.2646	0.025*	
C28	0.70451 (15)	0.50268 (16)	0.11784 (15)	0.0330 (5)	
H28A	0.7479	0.5619	0.0692	0.050*	
H28B	0.7199	0.4434	0.0968	0.050*	
H28C	0.7204	0.4874	0.1721	0.050*	
C29	0.33642 (15)	0.39742 (15)	0.17690 (13)	0.0279 (4)	
C51	−0.00963 (14)	0.68871 (15)	0.49817 (13)	0.0280 (4)	
H51A	−0.0012	0.6869	0.4385	0.042*	
H51B	−0.0443	0.7515	0.5069	0.042*	
H51C	−0.0528	0.6292	0.5466	0.042*	
C52	0.15263 (16)	0.58609 (15)	0.49100 (14)	0.0311 (4)	
H52A	0.1612	0.5824	0.4319	0.047*	
H52B	0.1087	0.5276	0.5404	0.047*	
H52C	0.2219	0.5845	0.4937	0.047*	
C53	0.08142 (17)	0.68508 (16)	0.59760 (13)	0.0334 (5)	
H53A	0.0394	0.6237	0.6436	0.050*	
H53B	0.0438	0.7461	0.6082	0.050*	
H53C	0.1496	0.6847	0.6020	0.050*	
C54	0.28570 (18)	0.44778 (18)	0.10799 (15)	0.0395 (5)	
H54A	0.3410	0.4757	0.0466	0.059*	
H54B	0.2415	0.5032	0.1248	0.059*	
H54C	0.2422	0.3967	0.1085	0.059*	
C55	0.25061 (18)	0.35823 (16)	0.27391 (14)	0.0403 (5)	
H55A	0.2834	0.3230	0.3172	0.060*	
H55B	0.2021	0.3106	0.2761	0.060*	
H55C	0.2116	0.4161	0.2904	0.060*	
C56	0.39518 (18)	0.30526 (17)	0.15303 (19)	0.0474 (6)	
H56A	0.4493	0.3285	0.0906	0.071*	
H56B	0.3453	0.2575	0.1573	0.071*	
H56C	0.4287	0.2708	0.1959	0.071*	
Si2	0.66618 (4)	0.86357 (4)	0.23586 (4)	0.02274 (12)	
Cl21	0.60060 (4)	0.96967 (4)	0.14308 (3)	0.03124 (12)	
Cl22	0.51693 (4)	0.80356 (4)	0.32911 (3)	0.03298 (12)	
O21	0.73042 (10)	0.81090 (9)	0.15725 (9)	0.0271 (3)	
O22	0.71224 (10)	0.96067 (9)	0.24424 (9)	0.0277 (3)	
N2	0.73095 (12)	0.76561 (11)	0.32095 (10)	0.0222 (3)	
C3	0.67413 (15)	0.75990 (15)	0.41947 (12)	0.0272 (4)	
H3A	0.6058	0.7226	0.4449	0.033*	
H3B	0.6587	0.8303	0.4211	0.033*	
C4	0.73222 (17)	0.70847 (17)	0.48170 (14)	0.0365 (5)	
H4A	0.6851	0.7000	0.5452	0.055*	
H4B	0.7940	0.7510	0.4639	0.055*	
H4C	0.7549	0.6413	0.4760	0.055*	
C31	0.79903 (14)	0.73245 (14)	0.14741 (13)	0.0246 (4)	
C32	0.79811 (15)	0.65335 (14)	0.22523 (13)	0.0242 (4)	
C33	0.86638 (15)	0.57413 (15)	0.21636 (14)	0.0288 (4)	
H33	0.8657	0.5203	0.2695	0.035*	
C34	0.93526 (16)	0.57225 (16)	0.13141 (15)	0.0323 (5)	
C35	0.93625 (15)	0.65319 (16)	0.05522 (15)	0.0321 (5)	
H35	0.9855	0.6528	−0.0032	0.039*	
C36	0.86891 (15)	0.73541 (15)	0.05932 (13)	0.0277 (4)	
C37	0.72758 (15)	0.65970 (14)	0.31734 (13)	0.0258 (4)	
H37A	0.7498	0.6101	0.3644	0.031*	
H37B	0.6543	0.6409	0.3315	0.031*	
C38	1.0096 (2)	0.4855 (2)	0.12239 (18)	0.0517 (7)	
H38A	1.0419	0.4709	0.1668	0.078*	
H38B	1.0647	0.5048	0.0604	0.078*	
H38C	0.9704	0.4244	0.1341	0.078*	
C39	0.87329 (16)	0.82352 (16)	−0.02677 (14)	0.0358 (5)	
C41	0.78091 (14)	0.97685 (14)	0.27893 (12)	0.0235 (4)	
C42	0.85004 (14)	0.90168 (14)	0.29709 (12)	0.0228 (4)	
C43	0.92354 (14)	0.91930 (14)	0.32735 (12)	0.0241 (4)	
H43	0.9717	0.8680	0.3389	0.029*	
C44	0.92693 (15)	1.01084 (15)	0.34079 (12)	0.0250 (4)	
C45	0.85391 (14)	1.08397 (14)	0.32416 (12)	0.0260 (4)	
H45	0.8550	1.1463	0.3347	0.031*	
C46	0.77966 (14)	1.07051 (14)	0.29293 (13)	0.0250 (4)	
C47	0.84438 (14)	0.80205 (14)	0.28365 (13)	0.0239 (4)	
H47A	0.8830	0.7501	0.3153	0.029*	
H47B	0.8784	0.8109	0.2177	0.029*	
C48	1.00732 (17)	1.03121 (16)	0.37228 (15)	0.0351 (5)	
H48A	1.0517	0.9719	0.3800	0.053*	
H48B	0.9710	1.0434	0.4308	0.053*	
H48C	1.0514	1.0916	0.3266	0.053*	
C49	0.70090 (17)	1.15270 (16)	0.27494 (16)	0.0369 (5)	
C61	0.76516 (17)	0.83455 (17)	−0.03338 (14)	0.0369 (5)	
H61A	0.7135	0.8543	0.0178	0.055*	
H61B	0.7425	0.7691	−0.0309	0.055*	
H61C	0.7707	0.8872	−0.0912	0.055*	
C62	0.9068 (2)	0.92360 (18)	−0.02516 (18)	0.0550 (7)	
H62A	0.8544	0.9399	0.0276	0.083*	
H62B	0.9120	0.9790	−0.0815	0.083*	
H62C	0.9755	0.9158	−0.0206	0.083*	
C63	0.9513 (2)	0.8040 (2)	−0.11370 (16)	0.0634 (8)	
H63A	1.0222	0.7984	−0.1138	0.095*	
H63B	0.9509	0.8609	−0.1674	0.095*	
H63C	0.9307	0.7402	−0.1157	0.095*	
C64	0.7148 (2)	1.24614 (18)	0.2974 (2)	0.0546 (7)	
H64A	0.7870	1.2744	0.2606	0.082*	
H64B	0.7013	1.2257	0.3625	0.082*	
H64C	0.6651	1.2981	0.2836	0.082*	
C65	0.7177 (2)	1.18947 (17)	0.17336 (18)	0.0509 (7)	
H65A	0.7886	1.2208	0.1354	0.076*	
H65B	0.6652	1.2400	0.1634	0.076*	
H65C	0.7101	1.1310	0.1567	0.076*	
C66	0.58778 (18)	1.1097 (2)	0.3360 (2)	0.0555 (7)	
H66A	0.5794	1.0830	0.4001	0.083*	
H66B	0.5737	1.0545	0.3186	0.083*	
H66C	0.5380	1.1642	0.3284	0.083*	
C71	0.4903 (3)	0.5234 (3)	0.5414 (3)	0.0369 (10)	0.50
C72	0.438 (2)	0.562 (2)	0.4837 (16)	0.040 (4)	0.50
H72	0.3895	0.6142	0.4923	0.048*	0.50
C73	0.4569 (4)	0.5248 (4)	0.4135 (4)	0.0494 (13)	0.50
H73	0.4198	0.5506	0.3746	0.059*	0.50
C74	0.5291 (11)	0.4508 (12)	0.4005 (9)	0.051 (3)	0.50
H74	0.5414	0.4246	0.3527	0.061*	0.50
C75	0.5841 (4)	0.4137 (4)	0.4556 (4)	0.0457 (12)	0.50
H75	0.6352	0.3633	0.4454	0.055*	0.50
C76	0.564 (2)	0.4509 (19)	0.5261 (16)	0.038 (3)	0.50
H76	0.6017	0.4258	0.5645	0.045*	0.50
C77	0.4664 (14)	0.5609 (13)	0.6206 (11)	0.054 (3)	0.50
H77A	0.4718	0.6358	0.5984	0.081*	0.50
H77B	0.5170	0.5333	0.6489	0.081*	0.50
H77C	0.3948	0.5380	0.6659	0.081*	0.50

### Atomic displacement parameters (Å^2^)

**Table:**

	*U*^11^	*U*^22^	*U*^33^	*U*^12^	*U*^13^	*U*^23^
Si1	0.0199 (3)	0.0228 (3)	0.0223 (3)	0.0003 (2)	−0.0087 (2)	−0.0071 (2)
Cl11	0.0228 (2)	0.0321 (3)	0.0331 (3)	−0.00531 (19)	−0.0087 (2)	−0.0119 (2)
Cl12	0.0303 (3)	0.0441 (3)	0.0354 (3)	−0.0001 (2)	−0.0197 (2)	−0.0029 (2)
O11	0.0252 (7)	0.0234 (7)	0.0240 (7)	−0.0061 (5)	−0.0042 (5)	−0.0085 (5)
O12	0.0206 (6)	0.0223 (7)	0.0300 (7)	−0.0006 (5)	−0.0086 (6)	−0.0104 (6)
N1	0.0214 (8)	0.0189 (8)	0.0196 (8)	0.0023 (6)	−0.0073 (6)	−0.0055 (6)
C1	0.0272 (10)	0.0248 (10)	0.0183 (9)	0.0016 (8)	−0.0081 (8)	−0.0045 (8)
C2	0.0277 (10)	0.0251 (10)	0.0238 (10)	0.0012 (8)	−0.0065 (8)	−0.0044 (8)
C11	0.0202 (9)	0.0210 (9)	0.0303 (10)	0.0028 (7)	−0.0105 (8)	−0.0111 (8)
C12	0.0196 (9)	0.0207 (9)	0.0265 (10)	0.0037 (7)	−0.0070 (8)	−0.0077 (8)
C13	0.0224 (9)	0.0202 (9)	0.0333 (11)	−0.0002 (7)	−0.0055 (8)	−0.0081 (8)
C14	0.0241 (10)	0.0262 (10)	0.0370 (11)	−0.0004 (8)	−0.0081 (9)	−0.0144 (9)
C15	0.0240 (10)	0.0288 (10)	0.0281 (10)	0.0013 (8)	−0.0092 (8)	−0.0124 (8)
C16	0.0184 (9)	0.0222 (9)	0.0260 (10)	0.0028 (7)	−0.0073 (8)	−0.0086 (8)
C17	0.0273 (10)	0.0169 (9)	0.0285 (10)	0.0021 (7)	−0.0083 (8)	−0.0049 (8)
C18	0.0478 (14)	0.0434 (13)	0.0445 (13)	−0.0174 (11)	−0.0081 (11)	−0.0216 (11)
C19	0.0222 (9)	0.0220 (9)	0.0237 (10)	0.0005 (7)	−0.0089 (8)	−0.0058 (8)
C21	0.0207 (9)	0.0227 (9)	0.0195 (9)	0.0008 (7)	−0.0083 (7)	−0.0053 (7)
C22	0.0245 (9)	0.0192 (9)	0.0172 (9)	0.0003 (7)	−0.0101 (7)	−0.0042 (7)
C23	0.0255 (9)	0.0222 (9)	0.0219 (9)	−0.0014 (7)	−0.0127 (8)	−0.0045 (7)
C24	0.0261 (10)	0.0239 (10)	0.0247 (10)	0.0029 (8)	−0.0140 (8)	−0.0074 (8)
C25	0.0303 (10)	0.0223 (9)	0.0278 (10)	0.0053 (8)	−0.0156 (8)	−0.0093 (8)
C26	0.0275 (10)	0.0210 (9)	0.0200 (9)	0.0000 (7)	−0.0115 (8)	−0.0057 (7)
C27	0.0229 (9)	0.0200 (9)	0.0211 (9)	−0.0001 (7)	−0.0106 (8)	−0.0050 (7)
C28	0.0288 (11)	0.0304 (11)	0.0469 (13)	0.0074 (9)	−0.0204 (10)	−0.0166 (10)
C29	0.0290 (10)	0.0263 (10)	0.0336 (11)	−0.0012 (8)	−0.0123 (9)	−0.0155 (9)
C51	0.0218 (9)	0.0303 (10)	0.0266 (10)	−0.0002 (8)	−0.0070 (8)	−0.0062 (8)
C52	0.0290 (11)	0.0256 (10)	0.0352 (11)	0.0019 (8)	−0.0118 (9)	−0.0075 (9)
C53	0.0397 (12)	0.0321 (11)	0.0271 (10)	−0.0046 (9)	−0.0139 (9)	−0.0057 (9)
C54	0.0392 (12)	0.0493 (14)	0.0411 (13)	−0.0034 (10)	−0.0201 (10)	−0.0213 (11)
C55	0.0443 (13)	0.0313 (12)	0.0376 (12)	−0.0132 (10)	−0.0078 (10)	−0.0082 (10)
C56	0.0367 (13)	0.0394 (13)	0.0801 (18)	0.0025 (10)	−0.0223 (13)	−0.0374 (13)
Si2	0.0236 (3)	0.0207 (3)	0.0264 (3)	0.0031 (2)	−0.0142 (2)	−0.0062 (2)
Cl21	0.0355 (3)	0.0273 (2)	0.0362 (3)	0.0078 (2)	−0.0241 (2)	−0.0069 (2)
Cl22	0.0254 (2)	0.0379 (3)	0.0346 (3)	−0.0002 (2)	−0.0151 (2)	−0.0064 (2)
O21	0.0324 (7)	0.0235 (7)	0.0259 (7)	0.0078 (6)	−0.0154 (6)	−0.0064 (6)
O22	0.0307 (7)	0.0213 (7)	0.0401 (8)	0.0049 (6)	−0.0249 (6)	−0.0087 (6)
N2	0.0232 (8)	0.0192 (8)	0.0252 (8)	0.0015 (6)	−0.0128 (7)	−0.0049 (6)
C3	0.0280 (10)	0.0287 (10)	0.0242 (10)	0.0009 (8)	−0.0117 (8)	−0.0065 (8)
C4	0.0407 (12)	0.0401 (12)	0.0295 (11)	−0.0002 (10)	−0.0198 (10)	−0.0047 (9)
C31	0.0244 (10)	0.0206 (9)	0.0340 (11)	0.0018 (7)	−0.0158 (8)	−0.0102 (8)
C32	0.0258 (10)	0.0209 (9)	0.0301 (10)	−0.0011 (7)	−0.0158 (8)	−0.0070 (8)
C33	0.0332 (11)	0.0248 (10)	0.0408 (12)	0.0041 (8)	−0.0257 (10)	−0.0128 (9)
C34	0.0294 (11)	0.0348 (11)	0.0472 (13)	0.0099 (9)	−0.0246 (10)	−0.0219 (10)
C35	0.0241 (10)	0.0414 (12)	0.0374 (12)	0.0035 (9)	−0.0129 (9)	−0.0205 (10)
C36	0.0227 (10)	0.0293 (10)	0.0326 (11)	−0.0012 (8)	−0.0121 (8)	−0.0102 (9)
C37	0.0306 (10)	0.0183 (9)	0.0288 (10)	0.0005 (8)	−0.0146 (8)	−0.0047 (8)
C38	0.0521 (15)	0.0574 (16)	0.0641 (17)	0.0314 (13)	−0.0354 (13)	−0.0357 (14)
C39	0.0305 (11)	0.0362 (12)	0.0301 (11)	−0.0016 (9)	−0.0053 (9)	−0.0058 (9)
C41	0.0233 (9)	0.0228 (9)	0.0257 (10)	0.0003 (7)	−0.0132 (8)	−0.0054 (8)
C42	0.0235 (9)	0.0202 (9)	0.0231 (9)	0.0011 (7)	−0.0098 (8)	−0.0048 (7)
C43	0.0220 (9)	0.0257 (10)	0.0239 (10)	0.0036 (8)	−0.0112 (8)	−0.0058 (8)
C44	0.0256 (10)	0.0296 (10)	0.0204 (9)	−0.0003 (8)	−0.0099 (8)	−0.0078 (8)
C45	0.0282 (10)	0.0241 (10)	0.0269 (10)	0.0011 (8)	−0.0104 (8)	−0.0104 (8)
C46	0.0243 (10)	0.0226 (10)	0.0268 (10)	0.0021 (8)	−0.0104 (8)	−0.0069 (8)
C47	0.0217 (9)	0.0226 (9)	0.0290 (10)	0.0035 (7)	−0.0140 (8)	−0.0063 (8)
C48	0.0380 (12)	0.0365 (12)	0.0437 (13)	0.0071 (9)	−0.0259 (10)	−0.0184 (10)
C49	0.0356 (12)	0.0267 (11)	0.0608 (15)	0.0110 (9)	−0.0281 (11)	−0.0209 (10)
C61	0.0422 (13)	0.0394 (12)	0.0266 (11)	0.0054 (10)	−0.0155 (10)	−0.0068 (9)
C62	0.0530 (15)	0.0389 (14)	0.0603 (17)	−0.0166 (12)	−0.0214 (13)	0.0029 (12)
C63	0.0490 (16)	0.075 (2)	0.0319 (13)	0.0121 (14)	0.0024 (12)	−0.0035 (13)
C64	0.0608 (16)	0.0366 (13)	0.095 (2)	0.0250 (12)	−0.0511 (16)	−0.0375 (14)
C65	0.0695 (17)	0.0269 (12)	0.0738 (18)	0.0110 (11)	−0.0537 (15)	−0.0094 (12)
C66	0.0309 (13)	0.0536 (16)	0.089 (2)	0.0167 (11)	−0.0228 (13)	−0.0372 (15)
C71	0.033 (2)	0.038 (3)	0.025 (2)	−0.009 (2)	−0.002 (2)	−0.002 (2)
C72	0.032 (6)	0.029 (5)	0.043 (8)	−0.003 (4)	−0.008 (4)	0.000 (4)
C73	0.042 (3)	0.057 (3)	0.037 (3)	−0.016 (3)	−0.018 (2)	0.006 (3)
C74	0.052 (5)	0.060 (6)	0.032 (7)	−0.024 (4)	0.000 (5)	−0.016 (4)
C75	0.036 (3)	0.034 (3)	0.055 (3)	0.001 (2)	−0.006 (2)	−0.015 (3)
C76	0.026 (5)	0.038 (7)	0.038 (6)	−0.004 (4)	−0.011 (4)	0.000 (4)
C77	0.060 (5)	0.054 (5)	0.032 (6)	−0.007 (4)	0.000 (4)	−0.017 (4)

### Geometric parameters (Å, º)

**Table:**

Si1—O11	1.6391 (13)	N2—C3	1.508 (2)
Si1—O12	1.6412 (13)	N2—C47	1.513 (2)
Si1—N1	2.0452 (15)	C3—C4	1.524 (3)
Si1—Cl12	2.0784 (7)	C3—H3A	0.9900
Si1—Cl11	2.1663 (7)	C3—H3B	0.9900
O11—C11	1.383 (2)	C4—H4A	0.9800
O12—C21	1.380 (2)	C4—H4B	0.9800
N1—C17	1.508 (2)	C4—H4C	0.9800
N1—C27	1.508 (2)	C31—C32	1.393 (3)
N1—C1	1.516 (2)	C31—C36	1.402 (3)
C1—C2	1.521 (3)	C32—C33	1.382 (3)
C1—H1A	0.9900	C32—C37	1.498 (3)
C1—H1B	0.9900	C33—C34	1.376 (3)
C2—H2A	0.9800	C33—H33	0.9500
C2—H2B	0.9800	C34—C35	1.387 (3)
C2—H2C	0.9800	C34—C38	1.509 (3)
C11—C12	1.389 (2)	C35—C36	1.400 (3)
C11—C16	1.398 (3)	C35—H35	0.9500
C12—C13	1.386 (3)	C36—C39	1.532 (3)
C12—C17	1.503 (3)	C37—H37A	0.9900
C13—C14	1.385 (3)	C37—H37B	0.9900
C13—H13	0.9500	C38—H38A	0.9800
C14—C15	1.395 (3)	C38—H38B	0.9800
C14—C18	1.509 (3)	C38—H38C	0.9800
C15—C16	1.396 (3)	C39—C63	1.530 (3)
C15—H15	0.9500	C39—C62	1.533 (3)
C16—C19	1.541 (2)	C39—C61	1.539 (3)
C17—H17A	0.9900	C41—C42	1.382 (2)
C17—H17B	0.9900	C41—C46	1.405 (3)
C18—H18A	0.9800	C42—C43	1.393 (3)
C18—H18B	0.9800	C42—C47	1.500 (2)
C18—H18C	0.9800	C43—C44	1.382 (3)
C19—C53	1.533 (3)	C43—H43	0.9500
C19—C51	1.538 (2)	C44—C45	1.397 (3)
C19—C52	1.543 (3)	C44—C48	1.510 (3)
C21—C22	1.386 (2)	C45—C46	1.393 (3)
C21—C26	1.405 (2)	C45—H45	0.9500
C22—C23	1.391 (2)	C46—C49	1.535 (3)
C22—C27	1.497 (2)	C47—H47A	0.9900
C23—C24	1.393 (3)	C47—H47B	0.9900
C23—H23	0.9500	C48—H48A	0.9800
C24—C25	1.387 (3)	C48—H48B	0.9800
C24—C28	1.506 (3)	C48—H48C	0.9800
C25—C26	1.396 (3)	C49—C64	1.532 (3)
C25—H25	0.9500	C49—C66	1.536 (3)
C26—C29	1.538 (3)	C49—C65	1.542 (3)
C27—H27A	0.9900	C61—H61A	0.9800
C27—H27B	0.9900	C61—H61B	0.9800
C28—H28A	0.9800	C61—H61C	0.9800
C28—H28B	0.9800	C62—H62A	0.9800
C28—H28C	0.9800	C62—H62B	0.9800
C29—C56	1.531 (3)	C62—H62C	0.9800
C29—C54	1.533 (3)	C63—H63A	0.9800
C29—C55	1.535 (3)	C63—H63B	0.9800
C51—H51A	0.9800	C63—H63C	0.9800
C51—H51B	0.9800	C64—H64A	0.9800
C51—H51C	0.9800	C64—H64B	0.9800
C52—H52A	0.9800	C64—H64C	0.9800
C52—H52B	0.9800	C65—H65A	0.9800
C52—H52C	0.9800	C65—H65B	0.9800
C53—H53A	0.9800	C65—H65C	0.9800
C53—H53B	0.9800	C66—H66A	0.9800
C53—H53C	0.9800	C66—H66B	0.9800
C54—H54A	0.9800	C66—H66C	0.9800
C54—H54B	0.9800	C71—C76	1.369 (15)
C54—H54C	0.9800	C71—C72	1.375 (15)
C55—H55A	0.9800	C71—C77	1.510 (11)
C55—H55B	0.9800	C72—C73	1.379 (14)
C55—H55C	0.9800	C72—H72	0.9500
C56—H56A	0.9800	C73—C74	1.368 (13)
C56—H56B	0.9800	C73—H73	0.9500
C56—H56C	0.9800	C74—C75	1.373 (13)
Si2—O22	1.6336 (13)	C74—H74	0.9500
Si2—O21	1.6386 (14)	C75—C76	1.381 (14)
Si2—N2	2.0440 (15)	C75—H75	0.9500
Si2—Cl22	2.0748 (7)	C76—H76	0.9500
Si2—Cl21	2.1797 (7)	C77—H77A	0.9800
O21—C31	1.378 (2)	C77—H77B	0.9800
O22—C41	1.381 (2)	C77—H77C	0.9800
N2—C37	1.507 (2)		
			
O11—Si1—O12	123.12 (7)	Cl22—Si2—Cl21	91.07 (3)
O11—Si1—N1	90.11 (6)	C31—O21—Si2	137.44 (12)
O12—Si1—N1	89.77 (6)	C41—O22—Si2	137.34 (12)
O11—Si1—Cl12	116.61 (5)	C37—N2—C3	107.61 (14)
O12—Si1—Cl12	120.27 (5)	C37—N2—C47	109.53 (14)
N1—Si1—Cl12	90.09 (5)	C3—N2—C47	110.89 (14)
O11—Si1—Cl11	89.23 (5)	C37—N2—Si2	108.63 (11)
O12—Si1—Cl11	89.23 (5)	C3—N2—Si2	113.74 (11)
N1—Si1—Cl11	178.24 (5)	C47—N2—Si2	106.38 (10)
Cl12—Si1—Cl11	91.66 (3)	N2—C3—C4	115.99 (16)
C11—O11—Si1	138.25 (12)	N2—C3—H3A	108.3
C21—O12—Si1	138.15 (11)	C4—C3—H3A	108.3
C17—N1—C27	108.95 (13)	N2—C3—H3B	108.3
C17—N1—C1	108.05 (13)	C4—C3—H3B	108.3
C27—N1—C1	109.98 (13)	H3A—C3—H3B	107.4
C17—N1—Si1	109.19 (11)	C3—C4—H4A	109.5
C27—N1—Si1	107.07 (10)	C3—C4—H4B	109.5
C1—N1—Si1	113.53 (11)	H4A—C4—H4B	109.5
N1—C1—C2	115.44 (15)	C3—C4—H4C	109.5
N1—C1—H1A	108.4	H4A—C4—H4C	109.5
C2—C1—H1A	108.4	H4B—C4—H4C	109.5
N1—C1—H1B	108.4	O21—C31—C32	119.12 (17)
C2—C1—H1B	108.4	O21—C31—C36	119.00 (16)
H1A—C1—H1B	107.5	C32—C31—C36	121.88 (17)
C1—C2—H2A	109.5	C33—C32—C31	119.73 (18)
C1—C2—H2B	109.5	C33—C32—C37	121.28 (17)
H2A—C2—H2B	109.5	C31—C32—C37	118.87 (16)
C1—C2—H2C	109.5	C34—C33—C32	120.80 (19)
H2A—C2—H2C	109.5	C34—C33—H33	119.6
H2B—C2—H2C	109.5	C32—C33—H33	119.6
O11—C11—C12	118.73 (16)	C33—C34—C35	118.25 (18)
O11—C11—C16	118.62 (16)	C33—C34—C38	120.4 (2)
C12—C11—C16	122.64 (17)	C35—C34—C38	121.3 (2)
C13—C12—C11	119.23 (17)	C34—C35—C36	123.85 (19)
C13—C12—C17	121.52 (16)	C34—C35—H35	118.1
C11—C12—C17	119.19 (16)	C36—C35—H35	118.1
C14—C13—C12	120.82 (17)	C35—C36—C31	115.47 (18)
C14—C13—H13	119.6	C35—C36—C39	122.00 (18)
C12—C13—H13	119.6	C31—C36—C39	122.53 (17)
C13—C14—C15	118.05 (17)	C32—C37—N2	111.59 (14)
C13—C14—C18	121.29 (18)	C32—C37—H37A	109.3
C15—C14—C18	120.65 (18)	N2—C37—H37A	109.3
C14—C15—C16	123.64 (18)	C32—C37—H37B	109.3
C14—C15—H15	118.2	N2—C37—H37B	109.3
C16—C15—H15	118.2	H37A—C37—H37B	108.0
C15—C16—C11	115.56 (16)	C34—C38—H38A	109.5
C15—C16—C19	122.07 (16)	C34—C38—H38B	109.5
C11—C16—C19	122.37 (16)	H38A—C38—H38B	109.5
C12—C17—N1	111.94 (14)	C34—C38—H38C	109.5
C12—C17—H17A	109.2	H38A—C38—H38C	109.5
N1—C17—H17A	109.2	H38B—C38—H38C	109.5
C12—C17—H17B	109.2	C63—C39—C36	111.58 (18)
N1—C17—H17B	109.2	C63—C39—C62	108.6 (2)
H17A—C17—H17B	107.9	C36—C39—C62	110.02 (18)
C14—C18—H18A	109.5	C63—C39—C61	106.17 (19)
C14—C18—H18B	109.5	C36—C39—C61	110.40 (16)
H18A—C18—H18B	109.5	C62—C39—C61	109.98 (19)
C14—C18—H18C	109.5	O22—C41—C42	119.30 (16)
H18A—C18—H18C	109.5	O22—C41—C46	118.72 (16)
H18B—C18—H18C	109.5	C42—C41—C46	121.96 (17)
C53—C19—C51	107.39 (15)	C41—C42—C43	119.83 (17)
C53—C19—C16	111.91 (15)	C41—C42—C47	119.06 (16)
C51—C19—C16	109.89 (15)	C43—C42—C47	121.11 (16)
C53—C19—C52	107.37 (16)	C44—C43—C42	120.57 (17)
C51—C19—C52	109.50 (15)	C44—C43—H43	119.7
C16—C19—C52	110.68 (15)	C42—C43—H43	119.7
O12—C21—C22	119.11 (16)	C43—C44—C45	118.02 (17)
O12—C21—C26	118.97 (15)	C43—C44—C48	120.94 (17)
C22—C21—C26	121.91 (16)	C45—C44—C48	121.03 (17)
C21—C22—C23	119.94 (16)	C46—C45—C44	123.61 (17)
C21—C22—C27	119.32 (16)	C46—C45—H45	118.2
C23—C22—C27	120.72 (16)	C44—C45—H45	118.2
C22—C23—C24	120.39 (17)	C45—C46—C41	115.97 (17)
C22—C23—H23	119.8	C45—C46—C49	122.25 (17)
C24—C23—H23	119.8	C41—C46—C49	121.78 (17)
C25—C24—C23	117.82 (17)	C42—C47—N2	110.54 (14)
C25—C24—C28	121.44 (17)	C42—C47—H47A	109.5
C23—C24—C28	120.74 (17)	N2—C47—H47A	109.5
C24—C25—C26	124.22 (17)	C42—C47—H47B	109.5
C24—C25—H25	117.9	N2—C47—H47B	109.5
C26—C25—H25	117.9	H47A—C47—H47B	108.1
C25—C26—C21	115.72 (16)	C44—C48—H48A	109.5
C25—C26—C29	121.76 (16)	C44—C48—H48B	109.5
C21—C26—C29	122.52 (16)	H48A—C48—H48B	109.5
C22—C27—N1	111.68 (14)	C44—C48—H48C	109.5
C22—C27—H27A	109.3	H48A—C48—H48C	109.5
N1—C27—H27A	109.3	H48B—C48—H48C	109.5
C22—C27—H27B	109.3	C64—C49—C46	111.23 (17)
N1—C27—H27B	109.3	C64—C49—C66	107.4 (2)
H27A—C27—H27B	107.9	C46—C49—C66	109.82 (18)
C24—C28—H28A	109.5	C64—C49—C65	107.58 (19)
C24—C28—H28B	109.5	C46—C49—C65	110.38 (18)
H28A—C28—H28B	109.5	C66—C49—C65	110.33 (19)
C24—C28—H28C	109.5	C39—C61—H61A	109.5
H28A—C28—H28C	109.5	C39—C61—H61B	109.5
H28B—C28—H28C	109.5	H61A—C61—H61B	109.5
C56—C29—C54	107.07 (17)	C39—C61—H61C	109.5
C56—C29—C55	108.17 (18)	H61A—C61—H61C	109.5
C54—C29—C55	109.91 (17)	H61B—C61—H61C	109.5
C56—C29—C26	111.70 (16)	C39—C62—H62A	109.5
C54—C29—C26	109.51 (16)	C39—C62—H62B	109.5
C55—C29—C26	110.41 (16)	H62A—C62—H62B	109.5
C19—C51—H51A	109.5	C39—C62—H62C	109.5
C19—C51—H51B	109.5	H62A—C62—H62C	109.5
H51A—C51—H51B	109.5	H62B—C62—H62C	109.5
C19—C51—H51C	109.5	C39—C63—H63A	109.5
H51A—C51—H51C	109.5	C39—C63—H63B	109.5
H51B—C51—H51C	109.5	H63A—C63—H63B	109.5
C19—C52—H52A	109.5	C39—C63—H63C	109.5
C19—C52—H52B	109.5	H63A—C63—H63C	109.5
H52A—C52—H52B	109.5	H63B—C63—H63C	109.5
C19—C52—H52C	109.5	C49—C64—H64A	109.5
H52A—C52—H52C	109.5	C49—C64—H64B	109.5
H52B—C52—H52C	109.5	H64A—C64—H64B	109.5
C19—C53—H53A	109.5	C49—C64—H64C	109.5
C19—C53—H53B	109.5	H64A—C64—H64C	109.5
H53A—C53—H53B	109.5	H64B—C64—H64C	109.5
C19—C53—H53C	109.5	C49—C65—H65A	109.5
H53A—C53—H53C	109.5	C49—C65—H65B	109.5
H53B—C53—H53C	109.5	H65A—C65—H65B	109.5
C29—C54—H54A	109.5	C49—C65—H65C	109.5
C29—C54—H54B	109.5	H65A—C65—H65C	109.5
H54A—C54—H54B	109.5	H65B—C65—H65C	109.5
C29—C54—H54C	109.5	C49—C66—H66A	109.5
H54A—C54—H54C	109.5	C49—C66—H66B	109.5
H54B—C54—H54C	109.5	H66A—C66—H66B	109.5
C29—C55—H55A	109.5	C49—C66—H66C	109.5
C29—C55—H55B	109.5	H66A—C66—H66C	109.5
H55A—C55—H55B	109.5	H66B—C66—H66C	109.5
C29—C55—H55C	109.5	C76—C71—C72	119.1 (11)
H55A—C55—H55C	109.5	C76—C71—C77	120.5 (9)
H55B—C55—H55C	109.5	C72—C71—C77	120.4 (10)
C29—C56—H56A	109.5	C71—C72—C73	120.6 (14)
C29—C56—H56B	109.5	C71—C72—H72	119.7
H56A—C56—H56B	109.5	C73—C72—H72	119.7
C29—C56—H56C	109.5	C74—C73—C72	119.3 (10)
H56A—C56—H56C	109.5	C74—C73—H73	120.4
H56B—C56—H56C	109.5	C72—C73—H73	120.4
O22—Si2—O21	123.90 (7)	C73—C74—C75	121.0 (8)
O22—Si2—N2	89.82 (6)	C73—C74—H74	119.5
O21—Si2—N2	90.50 (6)	C75—C74—H74	119.5
O22—Si2—Cl22	118.45 (6)	C74—C75—C76	118.9 (10)
O21—Si2—Cl22	117.65 (6)	C74—C75—H75	120.6
N2—Si2—Cl22	90.22 (5)	C76—C75—H75	120.6
O22—Si2—Cl21	89.46 (5)	C71—C76—C75	121.0 (13)
O21—Si2—Cl21	89.01 (5)	C71—C76—H76	119.5
N2—Si2—Cl21	178.71 (5)	C75—C76—H76	119.5
			
O12—Si1—O11—C11	91.20 (18)	O21—Si2—O22—C41	−80.39 (19)
N1—Si1—O11—C11	1.39 (18)	N2—Si2—O22—C41	10.09 (19)
Cl12—Si1—O11—C11	−88.76 (18)	Cl22—Si2—O22—C41	100.24 (18)
Cl11—Si1—O11—C11	179.77 (17)	Cl21—Si2—O22—C41	−168.83 (18)
O11—Si1—O12—C21	−77.28 (18)	O22—Si2—N2—C37	−167.49 (12)
N1—Si1—O12—C21	12.71 (17)	O21—Si2—N2—C37	−43.59 (12)
Cl12—Si1—O12—C21	102.68 (17)	Cl22—Si2—N2—C37	74.06 (11)
Cl11—Si1—O12—C21	−165.85 (17)	O22—Si2—N2—C3	72.70 (12)
O11—Si1—N1—C17	−43.24 (12)	O21—Si2—N2—C3	−163.40 (12)
O12—Si1—N1—C17	−166.36 (11)	Cl22—Si2—N2—C3	−45.75 (11)
Cl12—Si1—N1—C17	73.36 (11)	O22—Si2—N2—C47	−49.67 (11)
O11—Si1—N1—C27	74.56 (11)	O21—Si2—N2—C47	74.23 (11)
O12—Si1—N1—C27	−48.56 (11)	Cl22—Si2—N2—C47	−168.12 (10)
Cl12—Si1—N1—C27	−168.83 (10)	C37—N2—C3—C4	73.33 (19)
O11—Si1—N1—C1	−163.86 (12)	C47—N2—C3—C4	−46.4 (2)
O12—Si1—N1—C1	73.02 (12)	Si2—N2—C3—C4	−166.29 (14)
Cl12—Si1—N1—C1	−47.26 (11)	Si2—O21—C31—C32	24.3 (3)
C17—N1—C1—C2	75.05 (19)	Si2—O21—C31—C36	−154.78 (15)
C27—N1—C1—C2	−43.8 (2)	O21—C31—C32—C33	179.90 (16)
Si1—N1—C1—C2	−163.69 (12)	C36—C31—C32—C33	−1.1 (3)
Si1—O11—C11—C12	23.8 (3)	O21—C31—C32—C37	−4.0 (2)
Si1—O11—C11—C16	−155.50 (15)	C36—C31—C32—C37	174.98 (16)
O11—C11—C12—C13	178.62 (16)	C31—C32—C33—C34	−0.1 (3)
C16—C11—C12—C13	−2.1 (3)	C37—C32—C33—C34	−176.11 (17)
O11—C11—C12—C17	−4.4 (2)	C32—C33—C34—C35	1.7 (3)
C16—C11—C12—C17	174.91 (16)	C32—C33—C34—C38	179.93 (19)
C11—C12—C13—C14	−0.2 (3)	C33—C34—C35—C36	−2.1 (3)
C17—C12—C13—C14	−177.11 (17)	C38—C34—C35—C36	179.7 (2)
C12—C13—C14—C15	2.0 (3)	C34—C35—C36—C31	0.9 (3)
C12—C13—C14—C18	−179.20 (19)	C34—C35—C36—C39	179.66 (19)
C13—C14—C15—C16	−1.8 (3)	O21—C31—C36—C35	179.72 (16)
C18—C14—C15—C16	179.43 (19)	C32—C31—C36—C35	0.7 (3)
C14—C15—C16—C11	−0.3 (3)	O21—C31—C36—C39	1.0 (3)
C14—C15—C16—C19	179.62 (17)	C32—C31—C36—C39	−178.05 (17)
O11—C11—C16—C15	−178.42 (15)	C33—C32—C37—N2	133.06 (17)
C12—C11—C16—C15	2.3 (3)	C31—C32—C37—N2	−42.9 (2)
O11—C11—C16—C19	1.6 (3)	C3—N2—C37—C32	−169.87 (14)
C12—C11—C16—C19	−177.65 (16)	C47—N2—C37—C32	−49.23 (19)
C13—C12—C17—N1	135.31 (18)	Si2—N2—C37—C32	66.57 (16)
C11—C12—C17—N1	−41.6 (2)	C35—C36—C39—C63	5.0 (3)
C27—N1—C17—C12	−51.11 (19)	C31—C36—C39—C63	−176.3 (2)
C1—N1—C17—C12	−170.57 (15)	C35—C36—C39—C62	−115.6 (2)
Si1—N1—C17—C12	65.51 (16)	C31—C36—C39—C62	63.1 (2)
C15—C16—C19—C53	−1.7 (2)	C35—C36—C39—C61	122.8 (2)
C11—C16—C19—C53	178.25 (17)	C31—C36—C39—C61	−58.5 (2)
C15—C16—C19—C51	117.50 (19)	Si2—O22—C41—C42	17.2 (3)
C11—C16—C19—C51	−62.5 (2)	Si2—O22—C41—C46	−164.12 (15)
C15—C16—C19—C52	−121.43 (19)	O22—C41—C42—C43	176.50 (16)
C11—C16—C19—C52	58.5 (2)	C46—C41—C42—C43	−2.2 (3)
Si1—O12—C21—C22	11.8 (3)	O22—C41—C42—C47	−3.5 (3)
Si1—O12—C21—C26	−169.27 (13)	C46—C41—C42—C47	177.87 (17)
O12—C21—C22—C23	178.57 (15)	C41—C42—C43—C44	1.0 (3)
C26—C21—C22—C23	−0.3 (3)	C47—C42—C43—C44	−179.02 (17)
O12—C21—C22—C27	0.0 (2)	C42—C43—C44—C45	0.7 (3)
C26—C21—C22—C27	−178.91 (16)	C42—C43—C44—C48	−179.12 (18)
C21—C22—C23—C24	−0.2 (3)	C43—C44—C45—C46	−1.5 (3)
C27—C22—C23—C24	178.33 (16)	C48—C44—C45—C46	178.39 (18)
C22—C23—C24—C25	0.4 (3)	C44—C45—C46—C41	0.4 (3)
C22—C23—C24—C28	−179.64 (17)	C44—C45—C46—C49	−179.71 (18)
C23—C24—C25—C26	−0.1 (3)	O22—C41—C46—C45	−177.23 (16)
C28—C24—C25—C26	180.00 (18)	C42—C41—C46—C45	1.4 (3)
C24—C25—C26—C21	−0.5 (3)	O22—C41—C46—C49	2.9 (3)
C24—C25—C26—C29	179.11 (17)	C42—C41—C46—C49	−178.46 (18)
O12—C21—C26—C25	−178.25 (15)	C41—C42—C47—N2	−43.1 (2)
C22—C21—C26—C25	0.6 (3)	C43—C42—C47—N2	136.89 (17)
O12—C21—C26—C29	2.2 (3)	C37—N2—C47—C42	−173.05 (14)
C22—C21—C26—C29	−178.92 (16)	C3—N2—C47—C42	−54.44 (18)
C21—C22—C27—N1	−43.6 (2)	Si2—N2—C47—C42	69.72 (15)
C23—C22—C27—N1	137.84 (16)	C45—C46—C49—C64	−1.1 (3)
C17—N1—C27—C22	−173.96 (14)	C41—C46—C49—C64	178.8 (2)
C1—N1—C27—C22	−55.71 (18)	C45—C46—C49—C66	−119.9 (2)
Si1—N1—C27—C22	68.07 (15)	C41—C46—C49—C66	60.0 (3)
C25—C26—C29—C56	−2.1 (3)	C45—C46—C49—C65	118.3 (2)
C21—C26—C29—C56	177.42 (18)	C41—C46—C49—C65	−61.8 (2)
C25—C26—C29—C54	−120.56 (19)	C76—C71—C72—C73	3 (5)
C21—C26—C29—C54	59.0 (2)	C77—C71—C72—C73	−177.4 (18)
C25—C26—C29—C55	118.3 (2)	C71—C72—C73—C74	−1 (3)
C21—C26—C29—C55	−62.2 (2)	C72—C73—C74—C75	−1 (2)
O22—Si2—O21—C31	91.19 (18)	C73—C74—C75—C76	1 (2)
N2—Si2—O21—C31	1.08 (18)	C72—C71—C76—C75	−2 (5)
Cl22—Si2—O21—C31	−89.43 (18)	C77—C71—C76—C75	178.0 (18)
Cl21—Si2—O21—C31	179.88 (17)	C74—C75—C76—C71	0 (3)
