# Supplementary figures and images for: Crystal structure of 4,8-di-tert-butyl-6,6-di­chloro-13-ethyl-2,10-dimethyl-13,14-di­hydro-12H-dibenzo[d,i][1,3,7,2]dioxaza­silecine toluene 0.25-solvate
Source: Acta Crystallogr E Crystallogr Commun. 2015 Dec 16;71(Pt 12):o1065–6. doi: 10.1107/S2056989015023889 (PMC4719981; doi:10.1107/S2056989015023889)

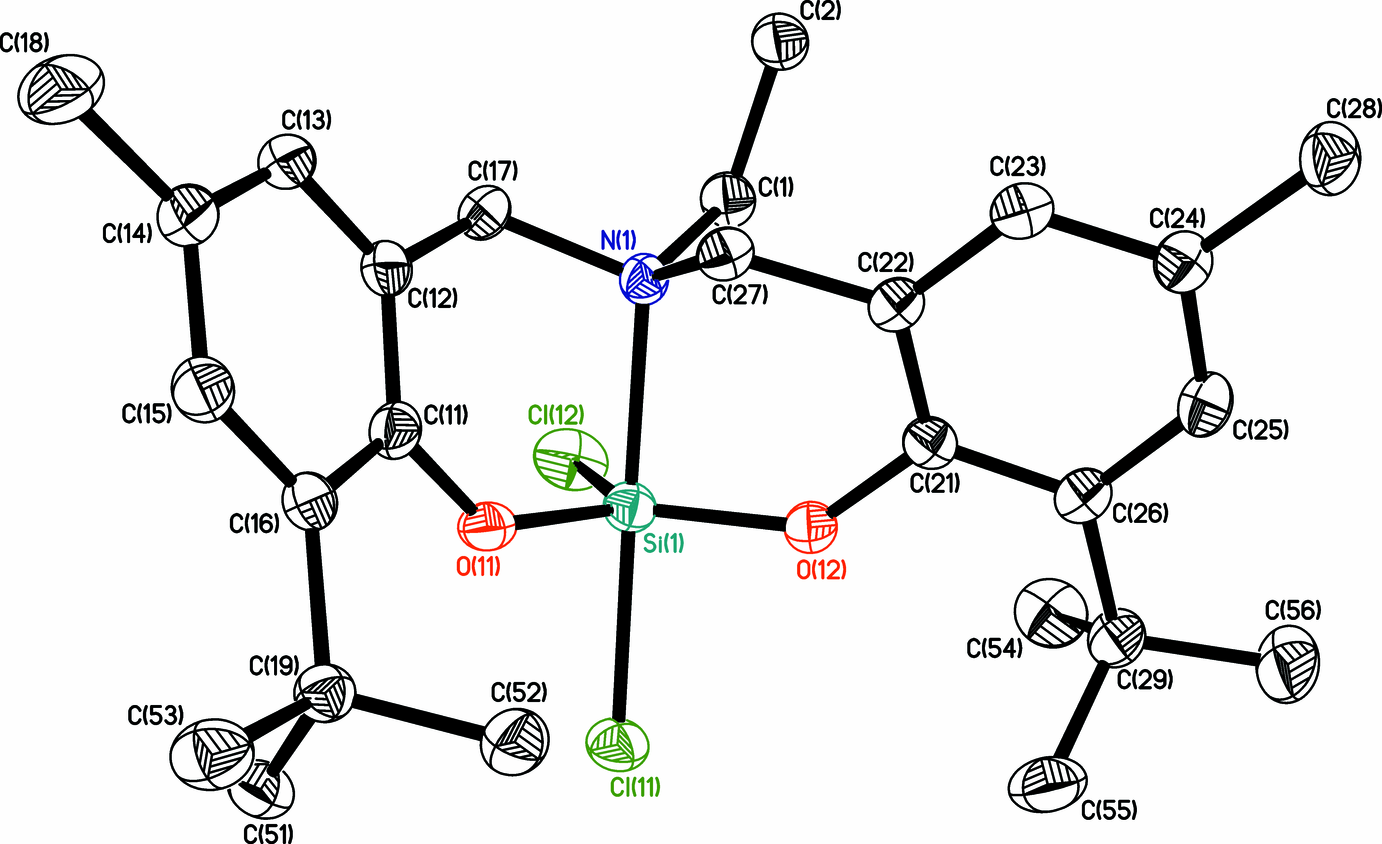

Supplement: Supplementary file 5 [file e-71-o1065-fig1.tif]
